# Supplementary material for: Anomalous quantum Griffiths singularity in ultrathin crystalline lead films
Source: Nat Commun. 2019 Aug 12;10:3633. doi: 10.1038/s41467-019-11607-w (PMC6690870; doi:10.1038/s41467-019-11607-w)
Supplement: Supplementary file 1 — Supplementary Information [file 41467_2019_11607_MOESM1_ESM.pdf]

Supplementary Information for

Anomalous quantum Griffiths singularity in ultrathin crystalline lead films

Liu and Wang et al.

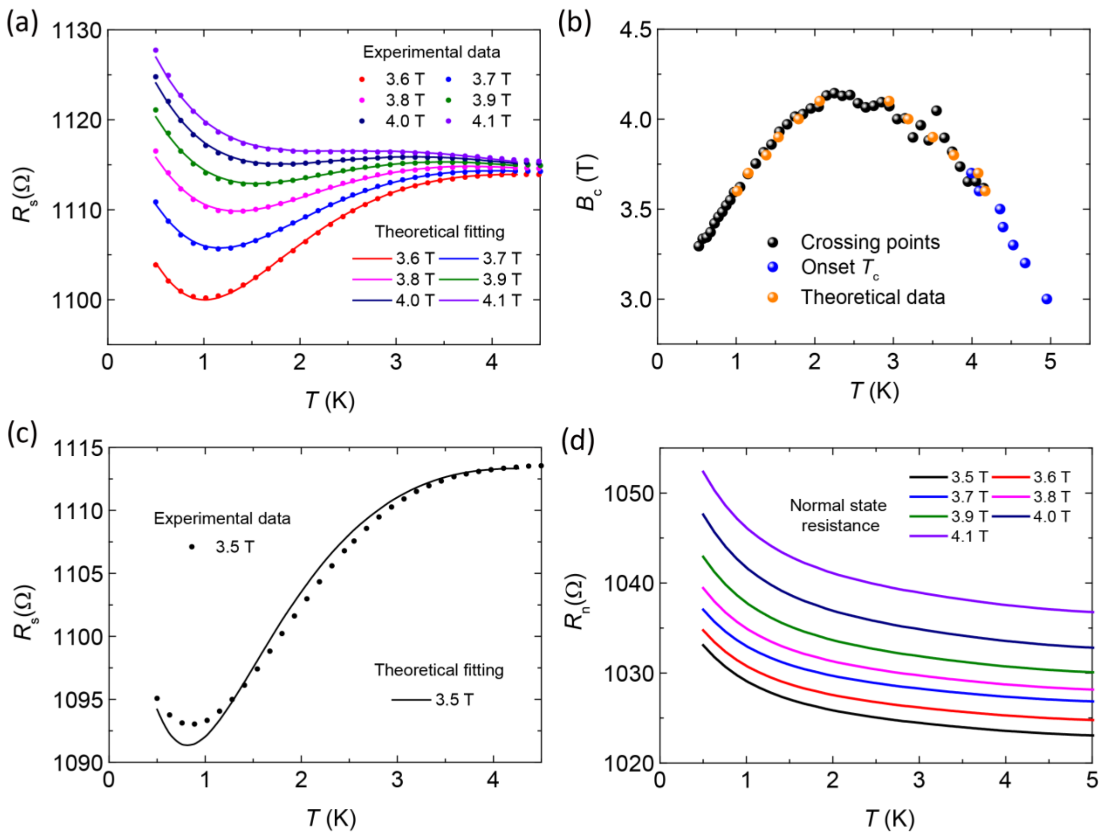

Supplementary Figure 1 | Theoretical analysis of the reentrant behavior in 4-ML Pb film. (a) The experimental $R_s(T)$ curves and the theoretical fitting curves (Supplementary Equation 4) of 4-ML Pb film from 3.6 T to 4.1 T. (b) The theoretical phase boundary from 3.6 T to 4.1 T, which is consistent with the experimental data. (c) The theoretical fitting curve deviates from the experimental $R_s(T)$ curve of 3.5 T, especially in the ultralow temperature regime, which may result from the influence of disorder. (d) The normal state resistance $R_n(T)$ curves at various magnetic fields for theoretical fitting. Note that the applied magnetic field is always perpendicular to the Pb films in this work.

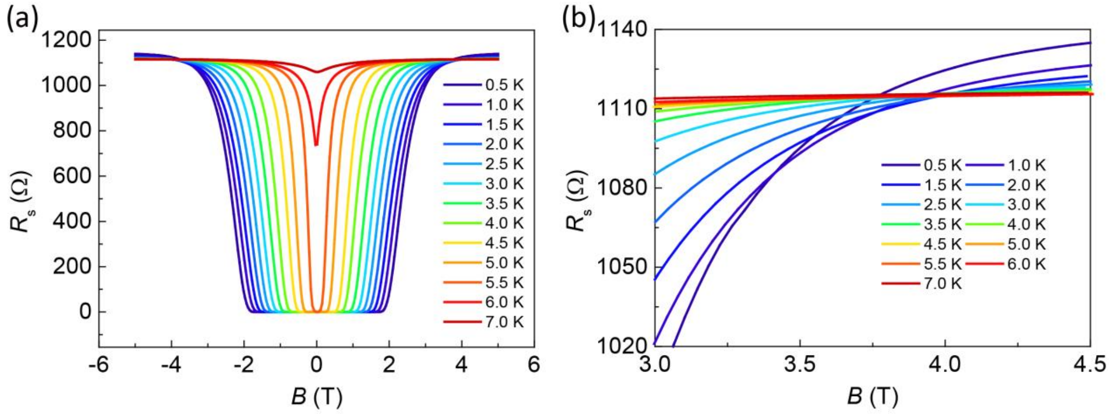

Supplementary Figure 2 | The magnetoresistance of 4-ML Pb film. (a) The magnetoresistance at different temperatures from 0.5 K to 7.0 K. (b) Close-up of the same data near the crossing region of $R_s(B)$ curves.

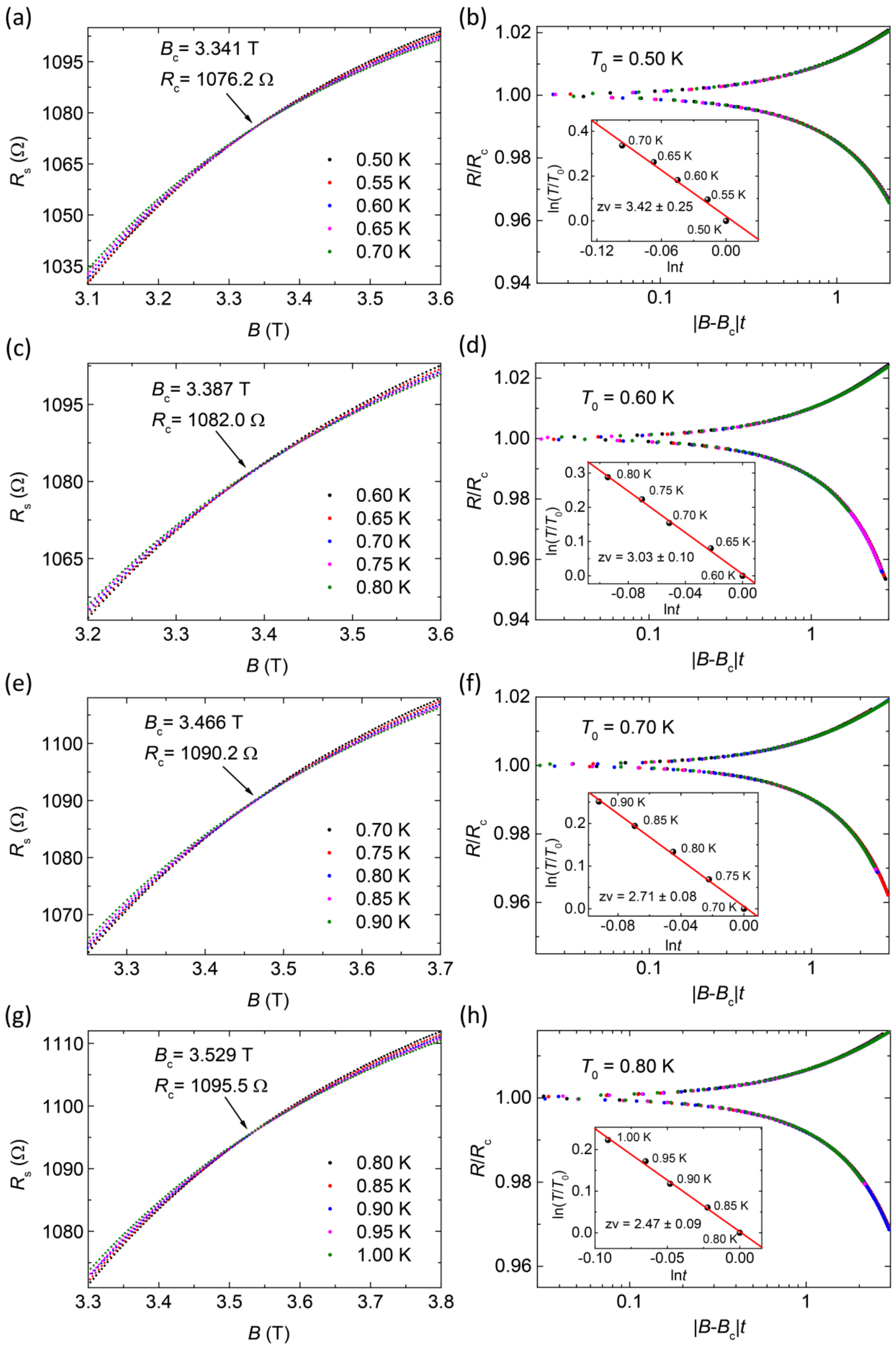

Supplementary Figure 3 | Finite-size scaling analysis for 4-ML Pb film at temperatures from 0.5 to 1.0 K. (a)(c)(e)(g) Sheet resistance as a function of magnetic field close to SMT boundary at various temperature ranges of (a) 0.5-0.7 K, (c) 0.6-0.8 K, (e) 0.7-0.9 K and (g) 0.8-1.0 K. (b)(d)(f)(h) Corresponding normalized sheet resistance as a function of scaling variable $|B - B_c|t$, with $t = T/T_0{}^{-1/z\nu}$. Inset: linear fitting between $\ln(T/T_0)$ and $\ln(t)$ gives critical exponent $z\nu$.

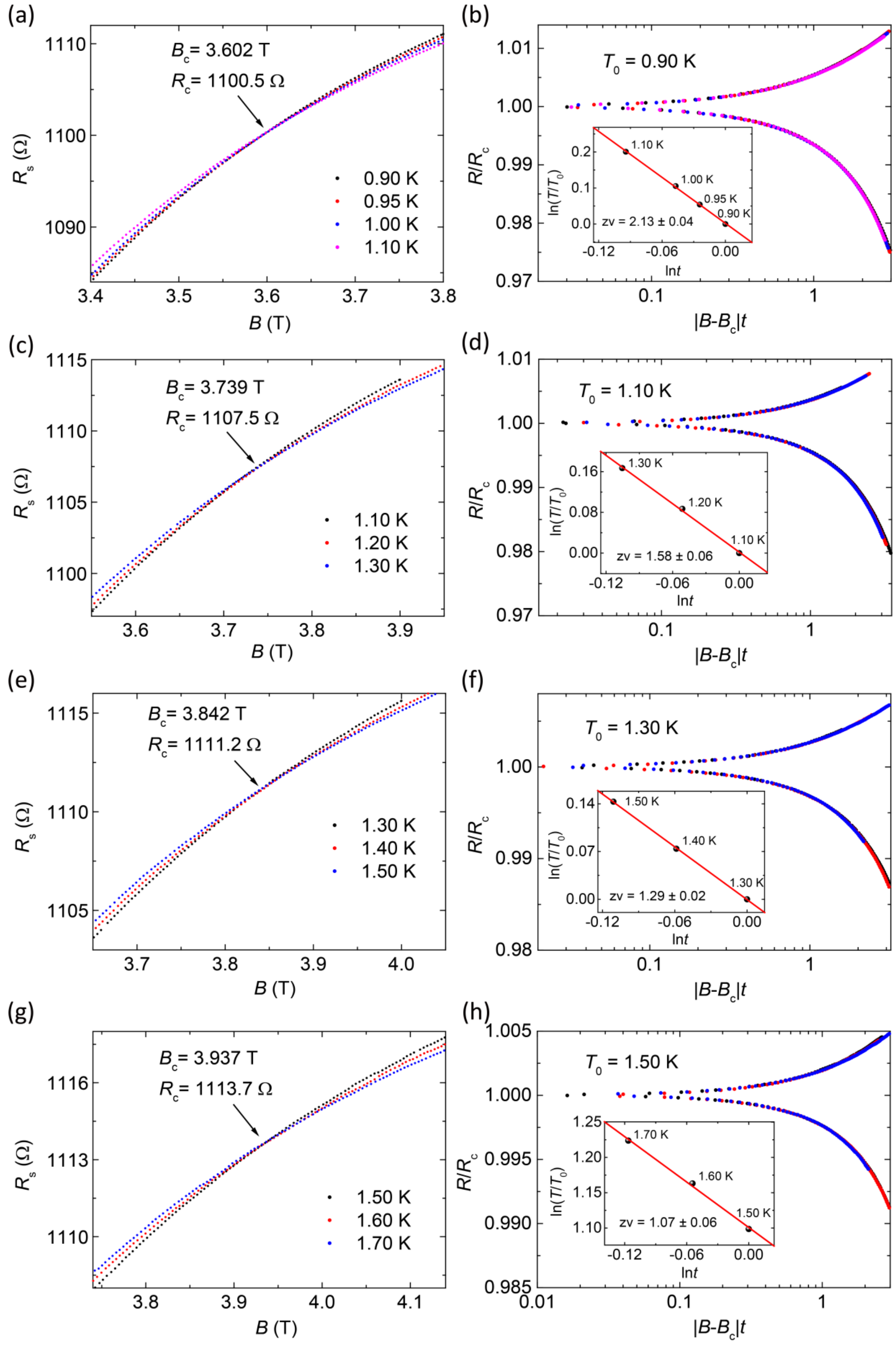

Supplementary Figure 4 | Finite-size scaling analysis for 4-ML Pb film at temperatures from 0.9 to 1.7 K. (a)(c)(e)(g) Sheet resistance as a function of magnetic field close to SMT boundary at various temperature ranges of (a) 0.9-1.1 K, (c) 1.1-1.3 K, (e) 1.3-1.5 K and (g) 1.5-1.7 K. (b)(d)(f)(h) Corresponding normalized sheet resistance as a function of scaling variable $|B - B_c|t$, with $t = T/T_0{}^{-1/z\nu}$. Inset: linear fitting between $\ln(T/T_0)$ and $\ln(t)$ gives critical exponent $z\nu$.

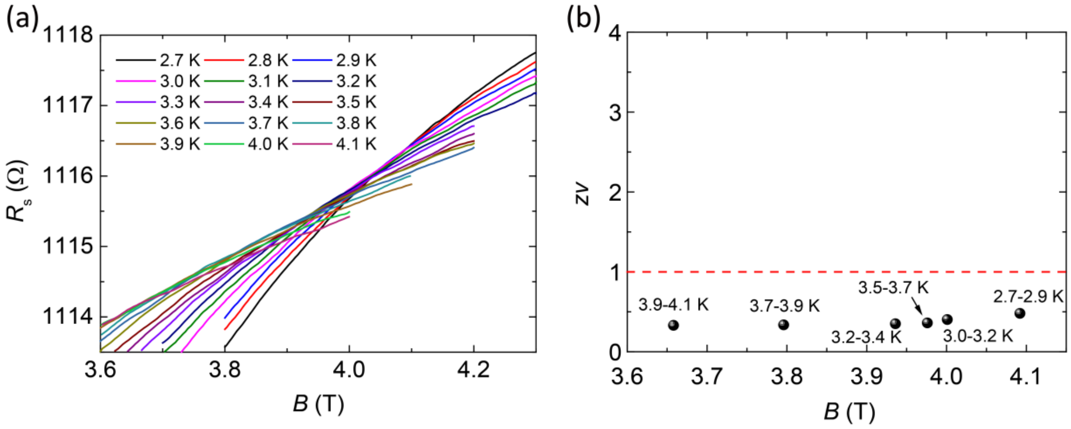

Supplementary Figure 5 | The magnetoresistance and the critical exponent $z\nu$ at relatively high temperatures. (a) The $R_s(B)$ curves at temperatures between 2.7 K and 4.1 K in 4-ML Pb film exhibit a pronounced transition region rather than a critical point. (b) Critical exponent $z\nu$ from 2.7 K to 4.1 K in 4-ML Pb film. In relatively high temperature regime, the $R_s(B)$ curves also exhibit a crossing region, but $z\nu$ is small (<1) and relatively stable.

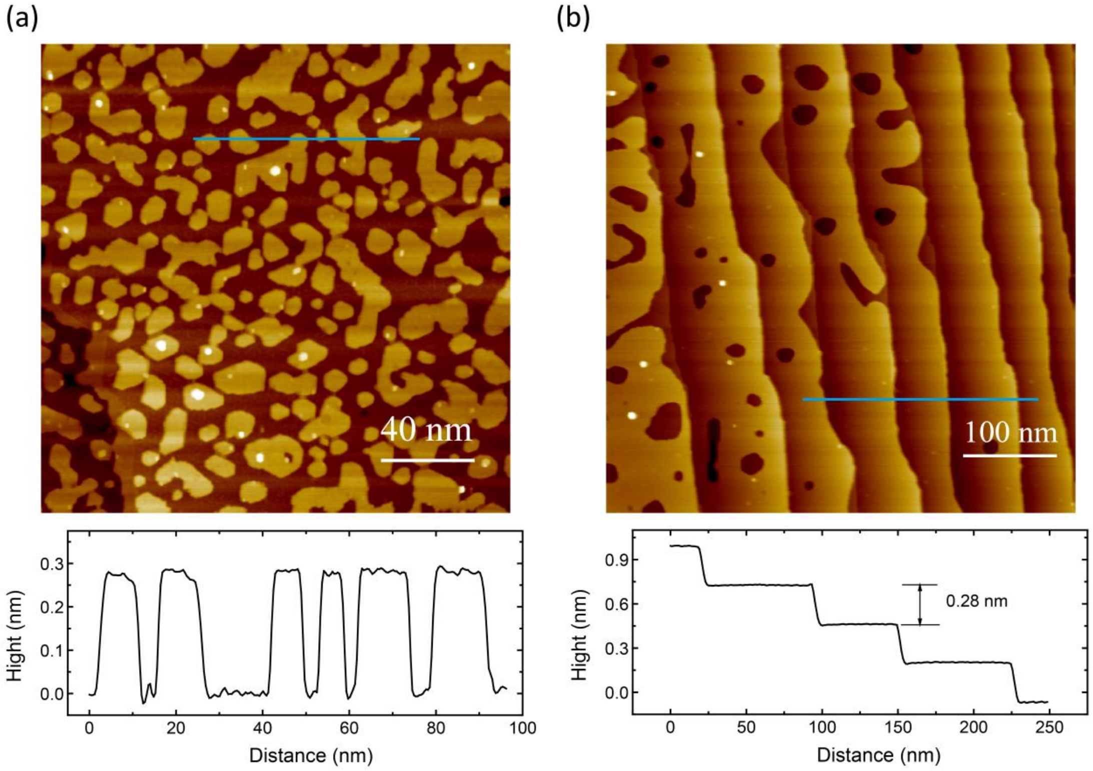

Supplementary Figure 6 | The morphology of ultrathin crystalline Pb films. Typical scanning tunneling microscopy images of (a) 3.5-ML and (b) 4-ML Pb film[1]. The thickness of the islands on 3.5-ML Pb film is one monolayer (around 0.28 nm).

Supplementary Table 1 | The parameters of Supplementary Equation 4 ($\sigma = \sigma_0 + d \cdot \sigma_{5T} + \frac{e^2}{\pi^2\hbar}\left[\alpha I_\alpha(b,t) + \beta I_\beta(b,t)\right] + C\left[\exp\left(-\frac{\Delta}{k_B T}\right) - 1\right]$) at different magnetic fields from 3.6 T to 4.1 T.

B (T)	α	β	C (Ω^{-1})	Δ/k_B (K)	$\sigma_0(\Omega^{-1})$	d
3.6	0.518	-0.233	9.71×10^{-5}	10.2	5.11×10^{-4}	0.519
3.7	0.518	-0.218	9.47×10^{-5}	10.3	5.00×10^{-4}	0.529
3.8	0.525	-0.205	9.33×10^{-5}	10.2	4.51×10^{-4}	0.583
3.9	0.527	-0.194	9.09×10^{-5}	10.2	3.80×10^{-4}	0.660
4.0	0.528	-0.195	8.72×10^{-5}	10.3	2.92×10^{-4}	0.755
4.1	0.515	-0.171	8.27×10^{-5}	10.3	2.57×10^{-4}	0.789

Supplementary Note 1. Superconducting fluctuation theory.

For two-dimensional superconductors in the dirty limit, the superconducting fluctuation correction to the conductivity is given by Larkin and his collaborators[2,3]. Based on recent full solution of superconducting fluctuation in two-dimensional systems without spin-orbit interaction (SOI) (*Phys. Rev.* B **84**, 104510 (2011)), the main contribution of superconducting fluctuation around B_{c2} (T=0) is the Maki-Thompson (MT) type term and diffusion coefficient renormalization (DCR) terms, which all relate to the Cooperon propagator. Meanwhile, in the ultrathin Pb films, there exists pronounced SOI, which gives rises to the Zeeman-protected superconductivity[1]. The SOI can alter the form of Cooperon propagator, introducing the spin-triplet channel with negative sign in addition to the spin-singlet channel with positive sign[4]. Thus, the values of α and β can be different from results in previous literatures[3,5]. In the following, we consider the influence of SOI on the Aslamazov-Larkin (AL) term and the MT-type terms (MT and DCR terms in the notation of Ref. 5), and the superconducting fluctuation effect on the conductivity correction can be written in the form[2,3]:

$$\delta\sigma = \frac{e^2}{\pi^2\hbar}[\alpha I_\alpha(b,t) + \beta I_\beta(b,t)] \tag{1}$$

with

$$I_\alpha(b,t) = \ln\frac{r}{b} - \frac{1}{2r} - \psi(r) \tag{2}$$

and

$$I_\beta(b,t) = r\psi'(r) - \frac{1}{2r} - 1, \tag{3}$$

where $r = \frac{b}{3.562t}$, $t = T/T_c \ll 1$, $b = [B - B_{c2}(T)]/B_{c2}(0) \ll 1$, $\psi(r)$ is the digamma function and $B_{c2}(T)$ is given by the Werthamer-Helfand-Hohenberg theory[6].

Furthermore, the superconducting fluctuation can decrease the density of states (DOS). In our simulation, we consider that the influence on the DOS can be represented by a thermal excitation of quasiparticle. In the high magnetic field regime (much larger than the superconducting critical field), the contribution of the

superconducting fluctuation becomes negligible and the temperature dependence of sheet resistance can represent the normal state resistance. Then we estimate the normal state conductance $\sigma_n = \sigma_0 + d \cdot \sigma_{5\mathrm{T}}$, where $\sigma_{5\mathrm{T}}$ is the sheet conductance of 4-ML Pb film at 5 T, and σ_0 and d are temperature independent parameters. Therefore, the total conductivity of the system can be written as:

$$\sigma = \sigma_n + \frac{e^2}{\pi^2\hbar}\left[\alpha I_\alpha(b,t) + \beta I_\beta(b,t)\right] + C\left[\exp\left(-\frac{\Delta}{k_B T}\right) - 1\right] \quad (4)$$

where Δ is the activation energy of thermal excitation (the local superconducting pairing strength). The experimental $R_s(T)$ curves from 3.6 T to 4.1 T can be well fitted by Supplementary Equation 4 (Supplementary Fig. 1) and the parameters are summarized in Supplementary Table 1. The normal state conductance is plotted in Supplementary Fig. 1(d), which exhibits the behavior of weakly localized metal state. And considering the effect of SOI on the AL term and MT-type terms (MT and DCR terms in the notation of Ref. 5), the parameters α and β are around 0.5 and -0.2, respectively, as shown in Supplementary Table 1. The values of α and β only slightly depend on the magnetic field, which accounts for the reliability of our consideration. For the DOS term, with increasing magnetic field from 3.6 T to 4.1 T, the local pairing strength Δ remains stable and the coefficient C decreases. It is reasonable that the influence of DOS term (this term takes into account the decrease of quasiparticle DOS due to formation of local fluctuating Cooper pairs) becomes smaller in higher magnetic field since the fluctuation Cooper pairs are also reduced.

Moreover, the minimum and maximum ($\mathrm{d}R/\mathrm{d}T = 0$) of conductivity correction (Supplementary Equation 4) from 3.6 T to 4.1 T give rise to the theoretical phase boundary since they separate the regions of $\mathrm{d}R/\mathrm{d}T < 0$ (weakly localized metal state) and $\mathrm{d}R/\mathrm{d}T > 0$ (superconducting state). Indeed, it is a common way to determine the phase boundary by the minima and maximum of the $R_s(T)$ curves[7]. As shown in Fig. 2(b) in the main text, the theoretical phase boundary above 3.6 T (corresponding to the temperatures above 1 K) is consistent with the experimental observations. Nevertheless, when approaching lower temperature and lower magnetic field regime along the phase boundary, the disorder induced higher order corrections beyond the

fluctuation effect become pronounced, which finally gives rise to the anomalous quantum Griffiths singularity. It is noteworthy to mention that the experimental $R_s(T)$ curve at 3.5 T begins to deviate from the theoretical formula of fluctuation effect (Supplementary Fig. 1(c)), especially at low temperatures below 1 K, which represents the higher order corrections induced by disorder effect when approaching the critical point. Thus, both the fluctuation effect and the disorder induced quantum Griffiths singularity are important to understand the SMT in our system.

The 4-ML Pb film has a relatively low mobility of 0.064 $cm^2\ V^{-1}\ s^{-1}$ in the low temperature regime (around 10 K), which is smaller than that of 3-ML Ga film (0.1 $cm^2\ V^{-1}\ s^{-1}$), 1-ML $NbSe_2$ film (30 $cm^2\ V^{-1}\ s^{-1}$) and $LaAlO_3/SrTiO_3(110)$ interface (112 $cm^2\ V^{-1}\ s^{-1}$) showing quantum Griffiths singularity. The low mobility in ultrathin Pb films enhances the effect of superconducting fluctuation.

Reentrant behaviors have been reported in superconductor-insulator/metal transition (SIT/SMT) of superconducting thin films[8,9]. We would like to point out two differences that distinguish our work from these earlier works. In the ultrathin Pb film, strong SOI can influence the parameter α and β of the superconducting fluctuation and hence change the extent of the superconducting fluctuations, which is different from previous studies on the reentrant behavior[8,9]. Furthermore, as shown in Ref. 8, the critical exponent $z\nu$ for the amorphous InO_x film is a constant around 1.3, indicating that the quantum Griffiths singularity is not observed. The homogenous disorder in amorphous InO_x film gives rise to SIT with critical resistance near $\frac{h}{4e^2}$, while in our case the disorder gives rise to non-homogenous rare region near the SMT with critical resistance much smaller than $\frac{h}{4e^2}$ and the rare regions are responsible for the quantum Griffiths singularity[10,11].

Last but not least, considering the influence of SOI, the explicit theoretical formula of the full solution of superconducting fluctuation might be derived in the similar procedure with previous literatures [3,5,12]. The analysis in our work may act as a phenomenological warm-up for the future in-depth investigation.

Supplementary Note 2. Discussions on quantum Griffiths singularity.

In the recent review paper[13], the authors suggest true quantum Griffiths state may not exist due to the reason that "Gapless degrees of freedom associated with the surrounding metallic state can only penetrate at most a distance ξ_0 into the cluster. Therefore, in the end, the coupling to the heat-bath can at most grow in proportion to the perimeter (surface area in 3D) of the cluster".

However, based on the Bardeen-Cooper-Schreiffer (BCS) theory, the superconducting coherence length $\xi_0 = \frac{\hbar v_F}{\pi\Delta(0)}$[14] (where $\Delta(0)$ is the superconducting gap and v_F is Fermi velocity) diverges with the superconducting gap $\Delta(0)$ tending to zero near the phase boundary[15]. This statement is valid along the entire phase boundary even when approaching zero temperature (the radius of the rare region keeps increasing and finally approaches infinite volume at this process). Therefore, it is possible to meet the requirement of quantum Griffiths singularity that ξ_0 should be larger than the scale of the rare regions. Moreover, in a recent in-depth investigation of two-dimensional superconducting fluctuation[5], the authors analyze the coherence cluster of fluctuating Cooper pairs with size $\xi_{QF} \sim \xi_{BCS}(H=0)/\sqrt{\frac{H_{c2}(0)}{H-H_{c2}(0)}}$, which diverges near the quantum critical point $H_{c2}(0)$ (Fig.4 and related discussion in Ref. 5) and thus is consistent with the prerequisite of quantum Griffiths singularity.

On the other hand, previous experiments on various 2D superconducting systems report $z\nu$ increases rapidly and tends to diverge with decreasing temperature in the ultralow temperature regime[11,16]. It is noteworthy to mention that the experimental value of $z\nu$ in these systems including the ultrathin Pb films changes in a large scale and does not have a trend to saturate even at the lowest temperatures achievable in dilution refrigerator, which can only be explained in the framework of quantum Griffiths singularity.

Supplementary References

1. Liu, Y. et al. Interface-induced Zeeman-protected superconductivity in ultrathin crystalline lead films. *Phys. Rev. X* **8**, 021002 (2018).
2. Aslamazov, L. G. & Larkin, A. I. Effect of fluctuations on properties of a superconductor above critical temperature. *Fiz. Tverd. Tela (Leningrad)* **10**, 1104 (1968).
3. Galitski, V. M. & Larkin, A. I. Superconducting fluctuations at low temperature. *Phys. Rev. B* **63**, 174506 (2001).
4. Bergmann, G. Weak localization in thin-films: a time-of-flight experiment with conduction electrons. *Phys. Rep.* **107**, 1-58 (1984).
5. Glatz, A., Varlamov, A. A. & Vinokur, V. M. Fluctuation spectroscopy of disordered two-dimensional superconductors. *Phys. Rev. B* **84**, 104510 (2011).
6. Werthame, N. R., Helfand, E. & Hohenber, P. C. Temperature and purity dependence of superconducting critical field Hc2. III. Electron spin and spin-orbit effects. *Phys. Rev.* **147**, 295 (1966).
7. Biscaras, J. et al. Multiple quantum criticality in a two-dimensional superconductor. *Nat. Mater.* **12**, 542-548 (2013).
8. Hebard, A. F. & Paalanen, M. A. Magnetic-field-tuned superconductor-insulator transition in 2-dimensional films. *Phys. Rev. Lett.* **65**, 927-930 (1990).
9. Hadacek, N., Sanquer, M. & Villegier, J. C. Double reentrant superconductor-insulator transition in thin TiN films. *Phys. Rev. B* **69**, 024505 (2004).
10. Vojta, T., Kotabage, C. & Hoyos, J. A. Infinite-randomness quantum critical points induced by dissipation. *Phys. Rev. B* **79**, 024401 (2009).
11. Xing, Y. et al. Quantum Griffiths singularity of superconductor-metal transition in Ga thin films. *Science* **350**, 542-545 (2015).
12. Varlamov, A. A., Galda, A. & Glatz, A. Fluctuation spectroscopy: From Rayleigh-Jeans waves to Abrikosov vortex clusters. *Rev. Mod. Phys.* **90**, 015009 (2018).
13. Kapitulnik, A., Kivelson, S. A. & Spivak, B. Colloquium: Anomalous metals: Failed superconductors. *Rev. Mod. Phys.* **91**, 011002 (2019).
14. Tinkham, M. *Introduction to superconductivity*. Courier Corporation (2004).
15. Cooper, L. N. *BCS: 50 years*. World scientific (2011), Chap. 11.

16. Saito, Y., Nojima, T. & Iwasa, Y. Quantum phase transitions in highly crystalline two-dimensional superconductors. *Nat. Commun.* **9**, 778 (2018).
